# Supplementary material for: Evidence supported by Mendelian randomization: impact on inflammatory factors in knee osteoarthritis
Source: Front Med (Lausanne). 2024 May 28;11:1382836. doi: 10.3389/fmed.2024.1382836 (PMC11165061; doi:10.3389/fmed.2024.1382836)
Supplement: Supplementary file 1 [file Data_Sheet_1.ZIP › data/GCST90274820_5e6/820.forest.pdf]

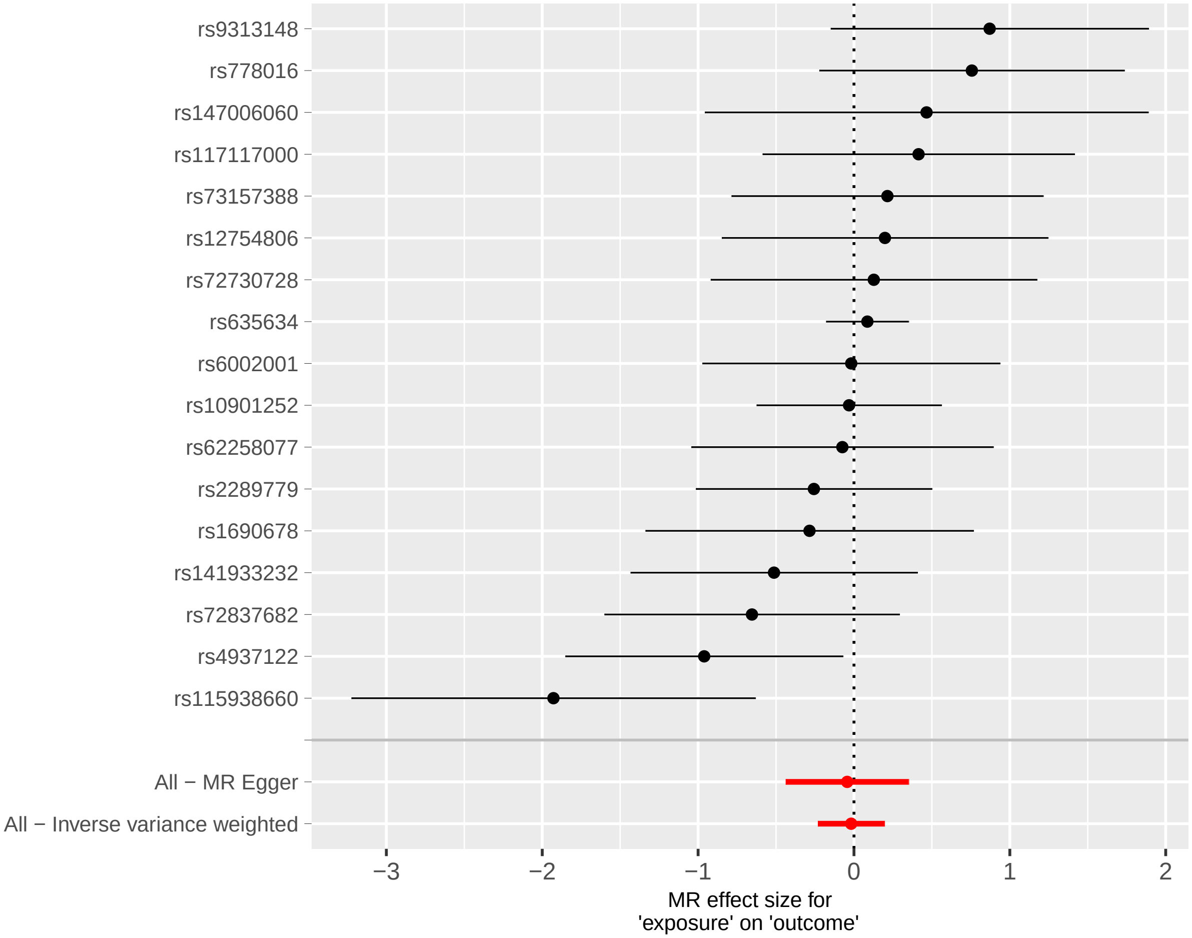

rs9313148
rs778016
rs147006060
rs117117000
rs73157388
rs12754806
rs72730728
rs635634
rs6002001
rs10901252
rs62258077
rs2289779
rs1690678
rs141933232
rs72837682
rs4937122
rs115938660
All − MR Egger
All − Inverse variance weighted
−3
−2
−1
0
1
2
MR effect size for
'exposure' on 'outcome'
